# Identification and High-Resolution Imaging of α-Tocopherol from Human Cells to Whole Animals by TOF-SIMS Tandem Mass Spectrometry

**DOI:** 10.1007/s13361-018-1979-x

**Published:** 2018-06-12

**Authors:** Anne L. Bruinen, Gregory L. Fisher, Rachelle Balez, Astrid M. van der Sar, Lezanne Ooi, Ron M. A. Heeren

**Affiliations:** 1 M4i, The Maastricht Multi Modal Molecular Imaging Institute, Maastricht University, 6229 ER Maastricht, The Netherlands; 2 Physical Electronics, Inc., Chanhassen, MN 55317 USA; 3 Illawarra Health and Medical Research Institute, School of Biological Sciences, University of Wollongong, Wollongong, NSW 2522 Australia; 4 VU University Medical Center Medical Microbiology and Infection control, 1081 HV Amsterdam, The Netherlands

**Keywords:** TOF-SIMS, Tandem MS, Mass spectrometry imaging, α-tocopherol, Vitamin E

## Abstract

A unique method for identification of biomolecular components in different biological specimens, while preserving the capability for high speed 2D and 3D molecular imaging, is employed to investigate cellular response to oxidative stress. The employed method enables observing the distribution of the antioxidant α-tocopherol and other molecules in cellular structures via time-of-flight secondary ion mass spectrometry (TOF-SIMS (MS^1^)) imaging in parallel with tandem mass spectrometry (MS^2^) imaging, collected simultaneously. The described method is employed to examine a network formed by neuronal cells differentiated from human induced pluripotent stem cells (iPSCs), a model for investigating human neurons in vitro. The antioxidant α-tocopherol is identified in situ within different cellular layers utilizing a 3D TOF-SIMS tandem MS imaging analysis. As oxidative stress also plays an important role in mediating inflammation, the study was expanded to whole body tissue sections of *M. marinum*-infected zebrafish, a model organism for tuberculosis. The TOF-SIMS tandem MS imaging results reveal an increased presence of α-tocopherol in response to the pathogen.

**Electronic supplementary material:**

The online version of this article (10.1007/s13361-018-1979-x) contains supplementary material, which is available to authorized users.

**Graphical Abstract:**
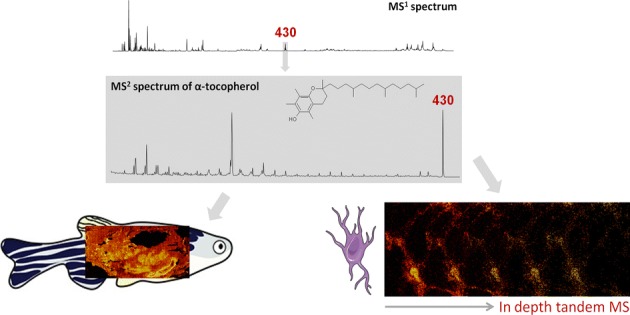
ᅟ

## Introduction

The brain is one of the major targets of lipid peroxidation, being highly sensitive to oxidative stress. This is a consequence of the high energy and oxygen demands of the brain, coupled with high levels of redox transition metals and polyunsaturated fatty acids, a common target of free radical attack. Resulting from lipid peroxidation, a variety of highly unstable and reactive free radical oxygen species are formed, which readily react with adjacent lipids, proteins, and nucleic acids to cause widespread disruption to neuronal homeostasis [1]. Markers of lipid peroxidation, such as malondialdehyde, which are F_2_- and F_4_-isoprostanes, are elevated in post mortem brain tissue of patients suffering from several neurodegenerative diseases including Alzheimer’s disease, amyotrophic lateral sclerosis, Huntington’s disease, and Parkinson’s disease [2–4]. Lipid-soluble vitamins, such as vitamin E, act as the first line of defense against oxidative insult in neurons, preserving membrane integrity and preventing the propagation of free radical-mediated damage [5]. Vitamin E is the collective term for α-, β-, γ-, and δ-tocopherol, of which α-tocopherol is the most prominent form in mammalian tissue. α-tocopherol is a lipid-soluble vitamin that serves as a potent antioxidant in cell membranes, offering protection by scavenging lipid peroxyl radicals through the donation of a hydrogen atom from the phenolic group on the chromanol ring [6]. Despite the vital role α-tocopherol holds in maintaining membrane health, unambiguous identification during imaging experiments has remained elusive.

In addition to neurodegenerative diseases, oxidative stress also plays an important role in mediating inflammation [7]. In a granulomatous tuberculosis infection, mycobacteria penetrate the host cells and are phagocytosed by macrophages trying to fight the infection. In this process, reactive oxygen species are generated, with the aim to kill the bacteria. This defense mechanism is usually unsuccessful, because of the antioxidant strategies of mycobacteria. Additionally, in severe infections, excess production of these reactive species results in inflammatory damage to the host tissue and can cause necrosis in the granuloma. For their part, the host tissue requires an antioxidative mechanism, with the aid of molecules such as α-tocopherol, to prevent damage from oxidative stress. In the search for a way to prevent and treat tuberculosis, visualizing regions susceptible to oxidative stress is crucial. For this, zebrafish (*Danio rerio)* infected with *Mycobacterium marinum* is a suitable and accepted model to study tuberculosis [8].

The difficulty of molecular identification is a lack of mass spectrometry imaging techniques that can simultaneously provide high mass resolution and high mass accuracy together with high spatial resolution and rapid molecular imaging. For analysis of inherently complex biological specimens, unambiguous identification of detected molecular species is essential. Until recently, biomolecular peaks observed by time-of-flight secondary ion mass spectrometry (TOF-SIMS) imaging have been identified based on the findings of published literature (under the assumption that those were correctly identified) or with the use of an ex situ analysis, e.g., matrix-assisted laser ablation desorption/ionization (MALDI) tandem mass spectrometry (MS/MS) [9–11].

A peak at *m*/*z* 430 is often observed in TOF-SIMS imaging of biological specimens. Monroe et al. identified an *m*/*z* 430 peak as α-tocopherol by comparison of their biological mass spectrum with a mass spectrum collected from a reference material. In their TOF-SIMS imaging study on *Aplysia california*, a common neurobiological model, they found co-localization of the *m*/*z* 430 molecular ion with two of the major fragments (*m*/*z* 165 and 205 of α-tocopherol that were also observed in the pure reference spectrum) [12]. Later, Passarelli et al. confirmed this finding with tandem MS [13]. In contrast, Altelaar et al. also observed a peak at *m*/*z* 430 in their TOF-SIMS imaging analysis of *Lymnaea stagnalis* nervous tissue, and linked that to the APGWamide neuropeptide with the aid of MALDI-MS/MS. The identity of the peak was confirmed with immunohistochemistry [14]. Neurological material was the subject of both studies, and very different conclusions were reached in each case. These examples highlight the need for a rapid, in situ capability for certain molecular identification that does not preclude the advantages of TOF-SIMS imaging which include a high repetition rate and < 100 nm lateral resolution imaging, high abundance sensitivity, no need for labelling or an applied ionization matrix, a shallow sampling depth, and analysis that does not consume the sample as is the case with MALDI imaging. Advances in instrumentation have resulted in innovative TOF-SIMS instruments with MS/MS capabilities that are now employed to address this limitation, in particular for biological applications. Examples include the implementation of a gallium liquid metal ion gun (LMIG) on a Fourier-transform ion cyclotron resonance (FT-ICR) mass spectrometer for high-resolution imaging combined with high mass resolution and MS/MS to achieve accurate identification [15], a C_60_ cluster ion source that was combined with FT-ICR to study larger biomolecules [16] along with several other tandem MS approaches [13, 17, 18]. The most recent development in this field is the 3D OrbiSIMS, presented by Passarelli and co-workers [19]. This hybrid instrument combines a time-of-flight analyzer with an orbitrap for 3D imaging combined with high mass resolution and tandem MS to achieve a comprehensive molecular insight in biological samples as small as a cell culture.

In this article, we demonstrate that with a single high-resolution analysis, collecting both TOF-SIMS (MS^1^) imaging and tandem MS (MS^2^) imaging data, one can directly arrive at the molecular identity of any targeted molecular precursor. High spatial resolution TOF-SIMS tandem MS imaging experiments were performed on a network of neuronal cells. Induced pluripotent stem cells (iPSCs) were used to generate human neurons in vitro, providing a useful tool for investigating cellular function, disease processes, and for drug screening or discovery [20, 21]. The analyzed iPSC-derived human neurons used in this study originated from a healthy donor. With the tandem MS imaging data, we were able to identify the *m*/*z* 430 peak as the molecular ion of α-tocopherol and map its distribution within substructures of the neuronal network. Furthermore, a tandem MS imaging experiment was conducted on whole body sections prepared from diseased and healthy zebrafish, where striking differences in the abundance of the peak at *m*/*z* 430 were observed. The infected and control fish specimens were part of a study on tuberculosis and, therefore, infected with *Mycobacterium marinum* as a model for human tuberculosis. Based on the product ions produced by collision-induced dissociation (CID), we identified the *m*/*z* 430 precursor as the molecular ion of α-tocopherol.

## Experimental Section

### Generation of Human iPSCs and Differentiation to Neurons

Fibroblasts from a 57-year-old healthy male were collected following methodology in accordance with guidelines set out in the National Statement on Ethical Conduct in Research Involving Humans (Australia), with informed consent obtained from the donor. The University of Wollongong Human Research Ethics Committee approved the experimental protocols used. The generation, maintenance, and characterization of human feeder-free iPSCs were carried out as previously described [22].

The iPSCs were differentiated to neurospheres, dissociated into single cells, and then differentiated to neurons as described previously [21, 22], with the following exception to optimally prepare the neurons for TOF-SIMS analysis: dissociated single cells were plated on silicon (Si) wafers (cut to 1 cm^2^) coated with collagen I (0.1 mg/mL, Life Technologies) and matrigel (In vitro Technologies), at a density of 20,000 cells per Si wafer piece. For culturing purposes, the Si wafers were placed in individual wells of a 24-well plate (GrienerBioOne).

All growth factors used for the final stages of neuronal differentiation were from Miltenyi Biotech (Bergisch Gladbach, Germany), except ascorbic acid and cyclic adenosine monophosphate which were from Sigma Aldrich (St. Louis, MO, USA). After overnight incubation, the growth medium was replaced by medium supplemented with 100 ng/mL fibroblast growth factor 8 (FGF8) and sonic hedgehog (SHH). On day 8 after plating, the medium was supplemented with 200 μM ascorbic acid, in addition to FGF-8 and SHH. On day 15, medium was supplemented with 100 ng/mL each of brain-derived neurotrophic factor, glial-derived neurotrophic factor, insulin-like growth factor 1, and cyclic adenosine monophosphate, in addition to SHH, FGF-8, and ascorbic acid until day 22. A partial medium change was performed every other day for the duration of the 22-day culture period.

### Cryofixation and Freeze-Drying of iPSC-Derived Neurons

On day 22, cells on Si wafers were removed from culture medium and gently rinsed in ammonium acetate (150 mM, brought to a pH of 7.4 using 1 M ammonium hydroxide (Sigma)) for 30 s, with excess liquid removed by blotting the edge on Kimwipes. The cells were then quickly submerged for 20 s into liquid nitrogen cooled liquid ethane and stored in liquid nitrogen until freeze-drying and imaging.

Prior to imaging, freeze-drying was performed in a FreeZone 2.5 L Benchtop Freeze Dry System (Labconco). The liquid nitrogen was drained from the falcon tubes containing Si wafers and the tubes quickly transferred into chambers pre-cooled to – 80 °C. The pressure was lowered to 0.014 mBar and the samples were freeze-dried overnight.

### Thin Tissue Sections of a Zebrafish Intestine Area

A healthy adult zebrafish (*D. rerio*) and an adult zebrafish infected by *M. marinum* (mutant strain mptC) as part of the study described by van der Sar et al. [23] were subjected to this TOF-SIMS experiment. Infection experiments were approved by the local Animal Welfare Committee, under protocol number MM01-02 and MM10-01 and all protocols adhered to the international guidelines specified by the EU Animal Protection Directive 86/609/EEC. The fish were chemically fixed in Dietrich’s fixative (30% ethanol, 10% formalin, 2% glacial acetic acid in deionized water). Prior to analysis, both fish were embedded in a mixture of 5% carboxymethyl cellulose and 10% gelatin (SigmaAldrich, Zwijndrecht, Netherlands) and frozen at – 80 °C as described in [24]. The blocks containing the fish were cut into 20-μm-thick sagittal sections with a cryo-microtome (Microm HM 525, Microm International, Walldorf, Germany). The thin tissue sections were thaw mounted on ITO-coated glass slides (Delta Technologies, Texas, USA) and stored at – 80 °C. The slides were dried and brought to ambient temperature in a desiccator approximately 30 min prior to analysis. Adjacent tissue sections were collected on Superfrost glass slides (Thermo Scientific, Waltham, MA, USA) to enhance attachment for histological staining. The slides were submerged in acetone at – 20 °C, before a sequence of 100%, 2 × 96%, and 2 × 70% ethanol baths. The tissues were stained with hematoxyline (Gill, Merck, Darmstadt, Germany) for 3 min, rinsed in tap water for 3 min, stained in a 0.2% eosin solution in ethanol (Klinipath, Breda, the Netherlands) for 30 s, rinsed in tap water for another minute, rinsed in 100% ethanol for a minute, and 30 s in xylol as the last step. Cover slips were mounted with Entellan (Merck, Darmstadt, Germany) and the slides were scanned with a Mirax digital slide scanner (Carl Zeiss, Sliedrecht, the Netherlands).

### TOF-SIMS Tandem MS Imaging

The TOF-SIMS imaging analyses were performed on a PHI *nanoTOF* II Parallel Imaging MS/MS instrument (Physical Electronics, Minnesota, USA), as discussed in references [17, 25]. The instrument was equipped with a 20k V argon gas cluster ion beam (GCIB) and a 30k V Bi_n_^q+^ cluster liquid metal ion gun (LMIG) for non-destructive sputtering and analysis, respectively. Briefly, after desorption, the secondary ions are extracted into a TRIFT spectrometer which consists of three electrostatic analyzers (ESAs). After the third ESA, an electrodynamic precursor selector is positioned so that the mass resolved ions can either fly through this crossover and be detected by the standard TOF-SIMS detector or be deflected into a collision cell for fragmentation. Generation of product ions is accomplished at approximately 1.5 keV in a collision-induced dissociation (CID) cell of Ar gas at a pressure of approximately 0.02 Pa. The product ions as well as the remaining precursor ions are then bunched and accelerated into a linear TOF analyzer before reaching the MS/MS detector. The precursor selection window is monoisotopic (i.e., 1 Da); however, a range of 3–4 Da on either side of the monoisotopic precursor selection window is perturbed. In each duty cycle, the deflected proportion of the precursor ions can be defined so that it is possible to maintain a portion of the precursor ions in the TOF-SIMS spectrum. At each image pixel, both TOF-SIMS (MS^1^) and tandem MS (MS^2^) data were simultaneously collected. Each pulse of the primary ion beam simultaneously triggers the time-of-flight clock in both spectrometers; hence, an exogenous calibration standard is not needed. The MS^1^ and MS^2^ time-of-flight was measured in 128 ps channels. Accounting for mass resolution, the accuracy of mass measurements in MS^1^ was 0.0049 Da at ca. *m*/*z* 50 and 0.0489 Da at ca. *m*/*z* 500; for MS^2^ measurements, the accuracy was 0.0260 Da and 0.1433 Da at ca. *m*/*z* 50 and ca. *m*/*z* 500, respectively. All images and spectra were produced retrospectively from the raw data files using PHI SmartSoft-TOF and PHI TOF-DR software (Physical Electronics, Minnesota, USA).

### Cells

A cell culture from human neurons was subjected to a conventional TOF-SIMS imaging experiment prior to an in-depth analysis: An MS^1^ spectrum of the sample surface was recorded for 10 frames, equal to 81 s, in positive ion mode using a mass pure beam of 30 keV Bi_3_^+^ ions having an unfiltered DC current of 12 nA. This survey analysis was conducted operating the primary ion beam in the HR^2^ mode, i.e., electrodynamically time compressed for high mass resolution while also achieving < 1 μm lateral resolution. A mass resolution of > 8800 (m/Δm_FWHM_) was measured at *m*/*z* > 250. The initial mass scale calibration was achieved using common fragments (C_2_H_5_^+^, *m*/*z* 29.0395; C_3_H_7_^+^, *m*/*z* 43.0553; C_4_H_9_^+^, *m*/*z* 57.0711) in the MS^1^ spectrum, and the precursor *m*/*z*, as observed in the MS^1^ spectrum, in the product ion spectrum. Improved mass accuracy at high *m*/*z* in the MS^1^ spectrum was achieved using ions identified by tandem MS as discussed thoroughly in the “Results and Discussion” section. In-depth characterization was executed in a 550 μm × 550 μm FOV containing 512 pixels × 512 pixels. Voxel imaging (i.e., 3D imaging depth profile) data was collected using a 5 keV beam of Ar_2,500_^+^ ions (5 nA DC beam current; rastered over an 800 μm × 800 μm area for 10 s between acquisition cycles of 20 frames (10.7 min) each using a mass pure beam of 30 keV Bi_3_^+^ ions (12 nA unfiltered DC beam current). For voxel imaging, the Bi_3_^+^ analytical ion beam was operated in the unbunched mode, i.e., not bunched for time compression, to achieve high lateral resolution. Hence, there was only unit mass resolution achieved in MS^1^; however, 2000–4000 mass resolution, measured as full width at half maximum at the precursor, was achieved in the MS^2^ spectrum together with high lateral resolution imaging as described elsewhere [25, 26]. The typical lateral resolution was measured to be below 700 nm but was also measured to go below 500 nm. The resulting beam doses per cycle were 6.86 × 10^11^ Bi_3_^+^/cm^2^ and 2.44 × 10^12^ Ar_2,500_^+^/cm^2^. Tandem MS imaging, with the precursor selection center at *m*/*z* 430.40, was performed concurrently with the TOF-SIMS 3D imaging.

### Tissue

Zebrafish thin tissue sections (healthy and infected) were characterized by a large area (mosaic) imaging analysis consisting of 32 × 16 image tiles, where each tile represented a 400 μm × 400 μm field-of-view (FOV), with 256 pixels × 256 pixels, and 5 imaging frames collected per tile. A mass pure beam of 30 keV Bi_3_^+^ ions, having an unfiltered DC current of 12 nA, was used to probe the tissue specimens in positive ion mode. The Bi_3_^+^ analytical ion beam was operated in the HR^2^ mode for analysis, i.e., electrodynamically time compressed for high mass resolution while also achieving < 1 μm lateral resolution. The mass resolution, full width at half maximum, was measured to be > 7000 at *m*/*z* > 250. The initial mass scale calibration was achieved using common fragments (C_2_H_5_^+^, *m*/*z* 29.0395; C_3_H_7_^+^, *m*/*z* 43.0553; C_4_H_9_^+^, *m*/*z* 57.0711) in the MS^1^ spectrum, and the precursor *m*/*z*, as observed in the MS^1^ spectrum, in the product ion spectrum. Improved mass accuracy at high *m*/*z* in the MS^1^ spectrum was achieved using ions identified by tandem MS as discussed in the “Results and Discussion” section. A primary ion dose density (PIDD) of 5.11 × 10^10^ Bi_3_^+^/cm^2^ was delivered to each 400 μm × 400 μm mosaic tile. Tandem MS imaging, with the precursor selection center at *m*/*z* 430.20, was performed subsequently on an area 300 μm × 300 μm in size for 200 acquisition frames, equal to 27 min and 2.73 × 10^12^ Bi_3_^+^/cm^2^.

## Results and Discussion

**Figure 1:**
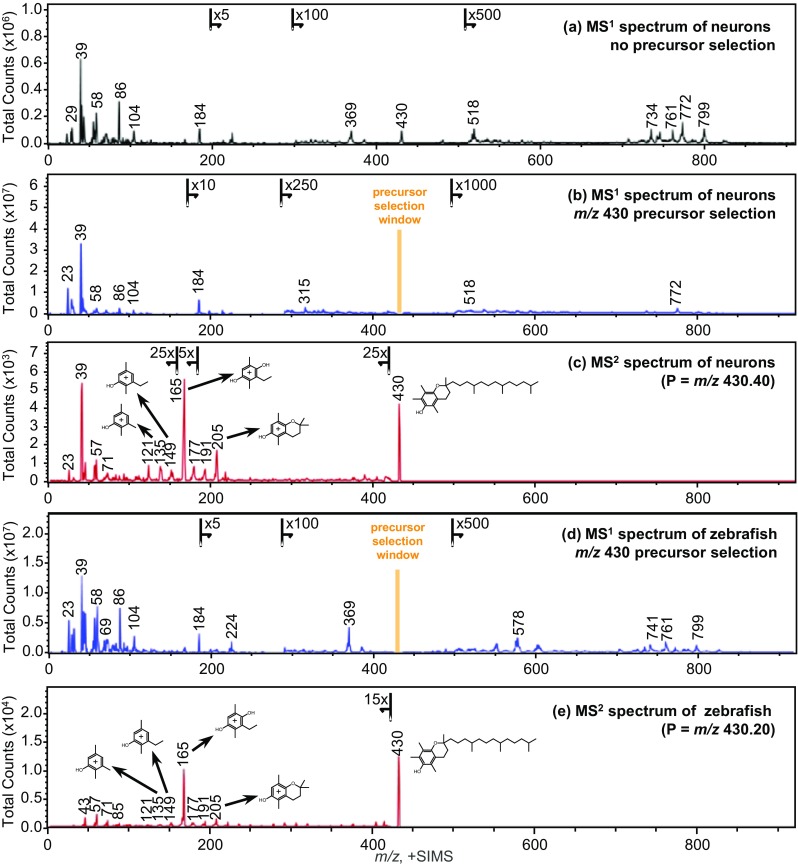
Mass spectra collected by TOF-SIMS tandem MS imaging in the positive ion mode, plotted in the range of *m*/*z* 0–920. (**a**) An MS^1^ precursor ion spectrum acquired from a cell culture of human neuronal cells prior to the 3D analysis. (**b**) The MS^1^ precursor ion spectrum summed over the entire 3D analysis. The *m/z* 430.40 precursor peak is no longer present. The orange marker highlights the distorted portion of the spectrum surrounding the monoisotopic precursor selection window. (**c**) The MS^2^ product ion spectrum, with precursor selection centered at *m/z* 430.40, summed over the entire 3D analysis. Some of the characteristic product ions, and the precursor, are annotated with their tentative structures. (**d**) An MS^1^ precursor ion spectrum acquired from a thin tissue section of zebrafish infected with *M. marinum* bacteria. The *m/z* 430.20 precursor peak is no longer present, and the orange marker highlights the distorted portion of the spectrum surrounding the monoisotopic precursor selection window. (**e**) The MS^2^ product ion spectrum, with precursor selection centered at *m*/*z* 430.20. Some of the characteristic product ions, and the precursor, are annotated with their tentative structures

A model human neural network was subjected to a simultaneous MS^1^ and MS^2^ experiment in order to explore the possibilities of the TOF-SIMS tandem MS imaging technique for molecular identification with high spatial resolution imaging. Figure 1(a) represents the MS^1^ spectrum of the cell culture surface before a precursor was selected for fragmentation in the CID cell. The peak at *m*/*z* 430 is highlighted and clearly visible in the overall spectrum. The TOF-SIMS tandem MS instrument allows partial deflection of a specific monoisotopic window into the collision cell and second TOF analyzer, but in the described experiment, 100% of the *m*/*z* 430.40 peak was selected so as to enhance the sensitivity. Sensitivity at high resolving power is a common challenge in small samples with high molecular complexity like cell cultures.

Figure 1(b) shows the MS^1^ spectrum acquired during an in-depth profiling experiment of 5 subsequent cycles of sputtering and analysis where *m*/*z* 430.40 was selected from the secondary ion stream and subjected to CID fragmentation. Hence, the depletion of the peak at *m*/*z* 430.40 in the MS^1^ spectrum. Figure 1(c) reveals the simultaneously acquired MS^2^ product ion spectrum. The peaks that lead to the identification of α-tocopherol by matching against product ion peaks found in the Scripps Metlin MS/MS metabolite database [27] include those at *m*/*z* 205, 191, 177, 165, 149, 136, 121, 71, 57, 43, and the *m*/*z* 430 precursor. The positive identification of the *m*/*z* 430.40 precursor as α-tocopherol using the Scripps Metlin MS/MS metabolite database was additionally confirmed using the NIST MS/MS reference library (Department of Commerce, Washington, D.C., USA) [28]. For each tandem MS reference library search, the nominal *m*/*z* of the significant product ions was used for the library matching. That is to say, neither the mass resolution nor the mass defect (i.e., mass accuracy) of the product ion peaks in the MS^2^ spectrum was a significant factor in achieving the initial reference library match. Subsequently, putative structures were assigned for the major product ions as revealed in Fig. 1(c); ions at *m*/*z* 71, 57, and 43 which are not labeled have compositions of C_5_H_11_^+^, C_4_H_9_^+^, and C_3_H_7_^+^, respectively. Further confidence in the precursor identification was achieved by exact mass following calibration of the product ion spectrum using the putative peak assignments; the resulting mass accuracies (Δ_*m/z*_) were measured to be +4.44 ppm across the product ion spectrum and +0.66 ppm at the precursor. These mass accuracies were determined with exclusion of the product ion peaks at *m*/*z* 23 and 39 and assuming that the majority of signal within the precursor selection window was that of α-tocopherol which seemed appropriate based on the reference library matches.

The fragmentation of the precursor ion at *m*/*z* 430.40 using 1.5 keV CID resembles the product ion spectra of the α-tocopherol [M]^+^ precursor in the Metlin and NIST databases. This strong similarity in the major product ions occurs independent of the different energy regimes or the spectrometer type. The Metlin reference library match was generated using an ESI Q-TOF instrument with 40 eV CID; the NIST reference library matches were generated by either an ESI Q-TOF instrument (Agilent, California, USA) using 27–45 eV CID, or an ESI LTQ instrument (Thermo Finnigan, California, USA) using 35% normalized collision energy CID. The aforementioned and other databases, including the human metabolite database [29], contain mainly low energy CID spectra. Besides the typical fragments of precursors found in these databases, additional bond cleavage is often observed in the high-energy CID spectra generated by TOF-SIMS tandem MS imaging. Fisher and colleagues [17] discussed such a spectrum of an erucamide standard as an example, showing neutral losses at each bond along the carbon chain, and including the easily identifiable double bond.

**Figure 2:**
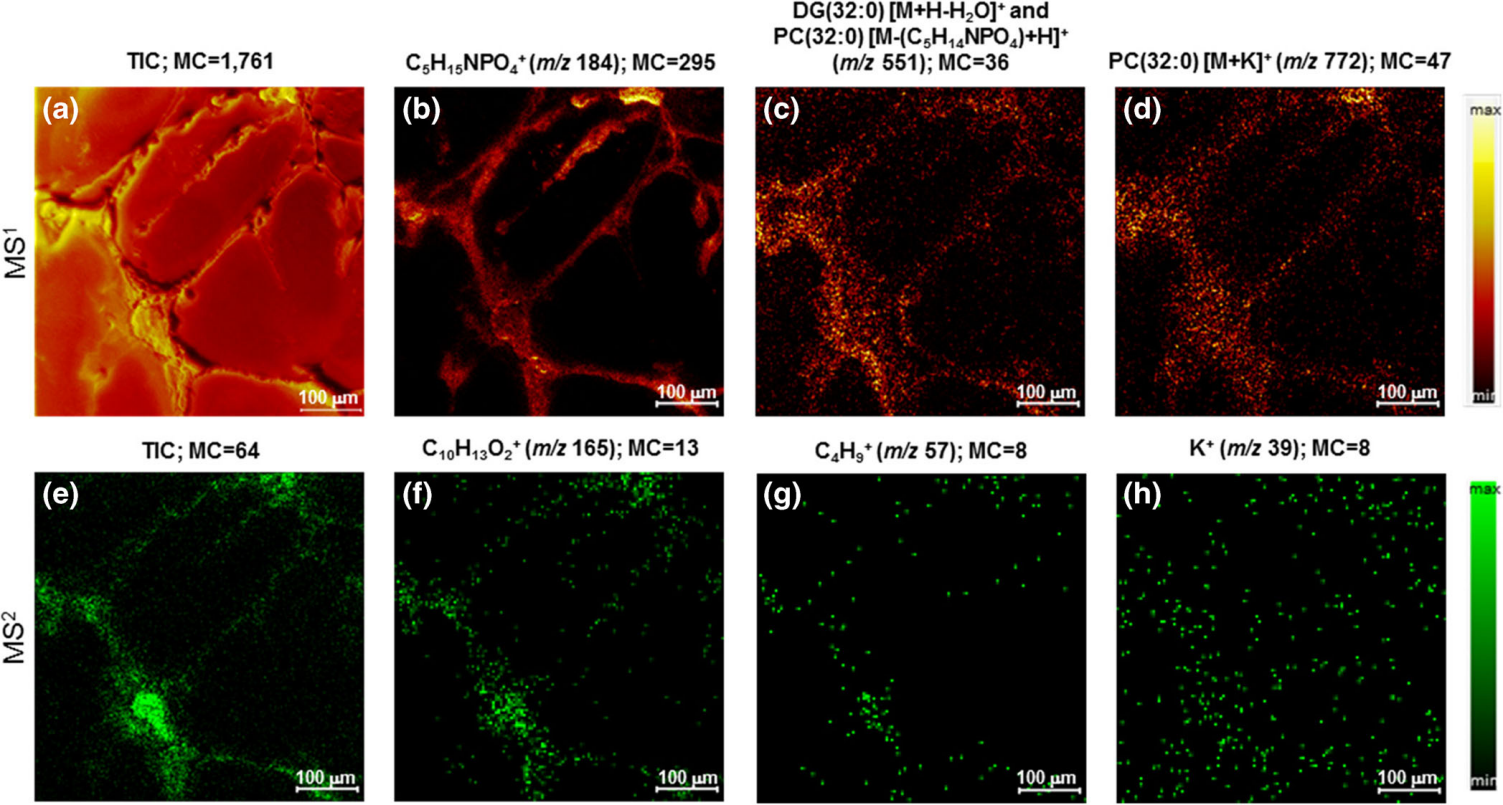
TOF-SIMS tandem MS imaging of the human iPSC-derived neurons in the positive ion mode. The top row are MS^1^ images and the bottom row are MS^2^ images which were collected simultaneously during the in-depth voxel imaging analysis. The maximum counts per pixel are given in each panel. The ion images in the top row include the total ion count (TIC) (**a**), phosphocholine at *m/z* 184 (**b**), presumed phospholipid fragment ion and/or a diacyl glycerol ion with a total fatty acid composition of (32:0) at *m/z* 551 (**c**), and presumed [M+K]^+^ ion of PC(32:0) at *m/z* 772 (**d**) produced from the MS^1^ data. The ion images in the bottom row include the TIC (all product ions and unfragmented precursor ions) of α-tocopherol (**e**), the prominent C_10_H_13_O_2_^+^ product ion of α-tocopherol at *m/z* 165 (**f**), a minor product ion of α-tocopherol at *m/z* 57 (**g**), and a K^+^ product ion at *m/z* 39 (**h**). Note that the K^+^ ions are not localized to the cells only, but are more disbursed throughout the image area

Apart from the characteristic peaks arising from α-tocopherol, appreciable signals were detected at *m*/*z* 23 and 39 which were attributed to Na^+^ and K^+^, respectively. The mass resolution in MS^2^ is adequate to observe K^+^ (*m*/*z* 38.9637) and NaO^+^ (*m*/*z* 38.9847) if they were both present, but only a single peak is present. The assignment of K^+^ at *m/z* 39 is based on the exact mass peak position subsequent to mass scale calibration without assuming a precursor composition in the calibration, i.e., letting the precursor exact mass float since there are, potentially, multiple precursor compositions. When using the NaO^+^ assignment the average mass accuracy (Δ_*m/z*_) was calculated in the software to be −3.31 ppm. The average mass accuracy (Δ_*m/z*_) was calculated to be +2.14 ppm, an absolute improvement of 1.17 ppm, when using the K^+^ assignment. Since the Na^+^ and K^+^ ions cannot originate from the fragmented α-tocopherol molecule, this observation is indicative of the fact that the monoisotopic precursor selection window contains multiple molecules. Clearly, the presence of the unresolved interference in the selection window centered at *m*/*z* 430.40 does not impede the identification of α-tocopherol. What is more, sodiated or potassiated molecular ions, i.e., [M+Na]^+^, typically produce characteristic product ions at much lower intensity than corresponding protonated molecular ions, i.e., [M+H]^+^. Therefore, it was not possible to identify the interference or interferences. The distribution of the K^+^ ions, as exposed in Fig. 2(h), is not limited to the neurons but, rather, is more homogeneously distributed within the image area. This observation, too, indicates an unresolved interference within the monoisotopic precursor selection window.

The TOF-SIMS tandem MS imaging method provides an additional benefit for improved molecular identification. We have stated earlier that no exogenous calibration reference is needed in MS^2^ because each pulse of the primary ion beam simultaneously triggers the time-of-flight clock in the MS^1^ and MS^2^ spectrometers (i.e., in the time-to-digital converter (TDC) electronics). Note, also, that the 52 μsec duty cycle of MS^2^ spectrometer is completed within the 120 μsec duty cycle of the MS^1^ spectrometer. Hence, we begin with the mass scale intercept and the precursor mass-to-charge ratio based on the MS^1^ timings. The product ion mass scale calibration may be enhanced with the addition of putative peak compositions. Compositional assignments may be judged as correct or incorrect based on their effect on the mass scale calibration, i.e., the sum of the mass deviations, as measured by the correlation coefficient (R) and the mass accuracy (Δ_*m/z*_). Ideally, one would like *R* > 0.99 and Δ_*m/z*-ave_ < 5 ppm. Importantly, and since the objective is to ascertain the identity of an unknown molecular precursor ion, high confidence is achieved if the mass accuracy at the precursor is < 1 ppm. In the case of the *m*/*z* 430.40 precursor ions arising from the neurons, we have identified the majority of ions within the precursor selection window as α-tocopherol [M]^+^ ions (C_29_H_50_O_2_^+^) to within a mass error of + 0.00028 Da (+ 0.66 ppm).

The molecular identification(s) can be returned to the MS^1^ spectrum for further data mining, and this is where the additional benefit of the TOF-SIMS tandem MS imaging comes forth. In the tandem MS analysis of the *m*/*z* 430.40 precursor ions, we identified not only the major precursor composition but also the product ion compositions. The ions identified in the product ion spectrum of α-tocopherol, e.g., C_13_H_17_O_2_^+^ (*m*/*z* 205.1241), C_10_H_13_O_2_^+^ (*m*/*z* 165.0925), and C_8_H_9_O^+^ (*m*/*z* 121.0660), can be imaged in TOF-SIMS and in tandem MS simultaneously, while the images produced by TOF-SIMS of these “small” ions may provide a stimulating visual effect that such images cannot be attributed to specific molecules renders them of little analytical value. Perhaps, the data garnered in MS^2^ can be leveraged in MS^1^ by other means.

The peak compositions identified in the MS^2^ spectrum can be included in the MS^1^ calibration with outstanding results. We begin this discussion having calibrated the MS^1^ spectrum shown in Fig. 1(a) using the ions of C_2_H_5_^+^ (*m*/*z* 29.0395), C_3_H_7_^+^ (*m*/*z* 43.0553), C_4_H_9_^+^ (*m*/*z* 57.0711), and C_5_H_15_NPO_4_^+^ (*m*/*z* 184.0749). We further begin this discussion, for the purpose of illustration, with the assumption that the peaks appearing at *m*/*z* 369.3555, *m*/*z* 385.3504, and *m*/*z* 772.5317 are identified as cholesterol [M+H-H_2_O]^+^, cholesterol [M-H]^+^ and PC(32:0) [M+K]^+^, respectively. With the MS^1^ calibration noted above, the centroid for each of these peaks are observed at *m/z* 369.3474 (Δ_*m/z*_ = 21.93 ppm), *m/z* 385.3356 (Δ_*m/z*_ = 38.41 ppm) and *m/z* 772.4677 (Δ_*m/z*_ = 82.84 ppm), respectively. Next, the same MS^1^ spectrum was calibrated using the ions of C_2_H_5_^+^ (*m/z* 29.0395), C_3_H_7_^+^ (*m/z* 43.0553), C_4_H_9_^+^ (*m/z* 57.0711), and C_29_H_50_O_2_^+^ (*m/z* 430.3848), the composition of α-tocopherol. The centroids of the cholesterol [M+H-H_2_O]^+^, cholesterol [M-H]^+^ and PC(32:0) [M+K]^+^ peaks are now observed at *m/z* 369.3555 (Δ_*m/z*_ = 0.00 ppm), *m/z* 385.3441 (Δ_*m/z*_ = 16.35 ppm), and *m/z* 772.5159 (Δ_*m/z*_ = 21.75 ppm), respectively, an improvement of up to 4× in the mass accuracy. In short, the direct identification(s) achieved by tandem MS analysis may be used to improve molecular classifications made by TOF-SIMS because the MS^1^ mass accuracy (Δ_*m/z*_) has been improved, in the present case from Δ_*m/z*_ > ca. 40 ppm to Δ_*m/z*_ < ca. 20 ppm at several hundred Da removed from the peak of MS^2^ characterization. To be sure, the aforementioned peak assignments (e.g., at *m/z* 369.3555, *m/z* 385.3441, and *m/z* 772.5159) require that tandem MS analyses be conducted for identification. Nevertheless, tentative peak assignments or molecular classifications can be enhanced using the information garnered from the tandem MS analysis of other molecules, in the present case that of α-tocopherol.

The distribution represented in the MS^2^ total ion count, which includes all product ions and unfragmented precursor ions in the MS^2^ spectrum generated from the *m/z* 430.40 precursor ions deflected from the MS^1^ spectrum, is plotted in Fig. 2(e). The MS^2^ TIC image exposes the iPSC-derived neuronal network, including the soma and the thread-like neurites. The distribution of the most prominent product ion at *m/z* 165, and of a minor product ion at *m/z* 57, are disclosed in Fig. 2(f) and (g). These tandem MS images, having high signal-to-noise, illustrate that α-tocopherol occurs at high abundance in the soma but is also appreciable in the neurites of healthy neurons.

A great advantage of the TOF-SIMS tandem MS imaging design is that all MS^1^ ions are retained during the selection and fragmentation of any precursor ion. This feature enabled us to investigate the distribution not only of α-tocopherol but of other lipid species that could potentially play a role in the formation and activity of neurons and their neurites. As an example, the distribution of ions at *m/z* 772and *m/z* 551 are highlighted in Fig. 2(c) and (d). These species were previously designated as the [M+K]^+^ ion of PC(32:0) and its presumable fragment after neutral loss of the phosphocholine headgroup at *m/z* 772and 551, respectively. No further characterization by TOF-SIMS tandem MS imaging was performed during the 3D analysis, but inclusion of the α-tocopherol composition in the MS^1^ calibration has boosted confidence in the assertion that the *m/z* 772.is likely the [M+K]^+^ ion of PC(32:0). Nevertheless, the slightly differing distributions of the *m/z* 772 and the *m/z* 551 ions argue for the fact that the latter is not exclusively a PC(32:0) fragment, but is in part comprised of another lipid component. This additional lipid component appears to be more prevalent in or near the plasma membrane of the cell. Notice specifically that the *m/z* 551 ions are enhanced at the outer perimeter of the soma and the neurites, while the *m/z* 772 ions are more evenly disbursed within the soma and are more localized to the central axes of the neurites. We surmise that this additional component at *m/z* 551 is DG(32:0).

**Figure 3:**
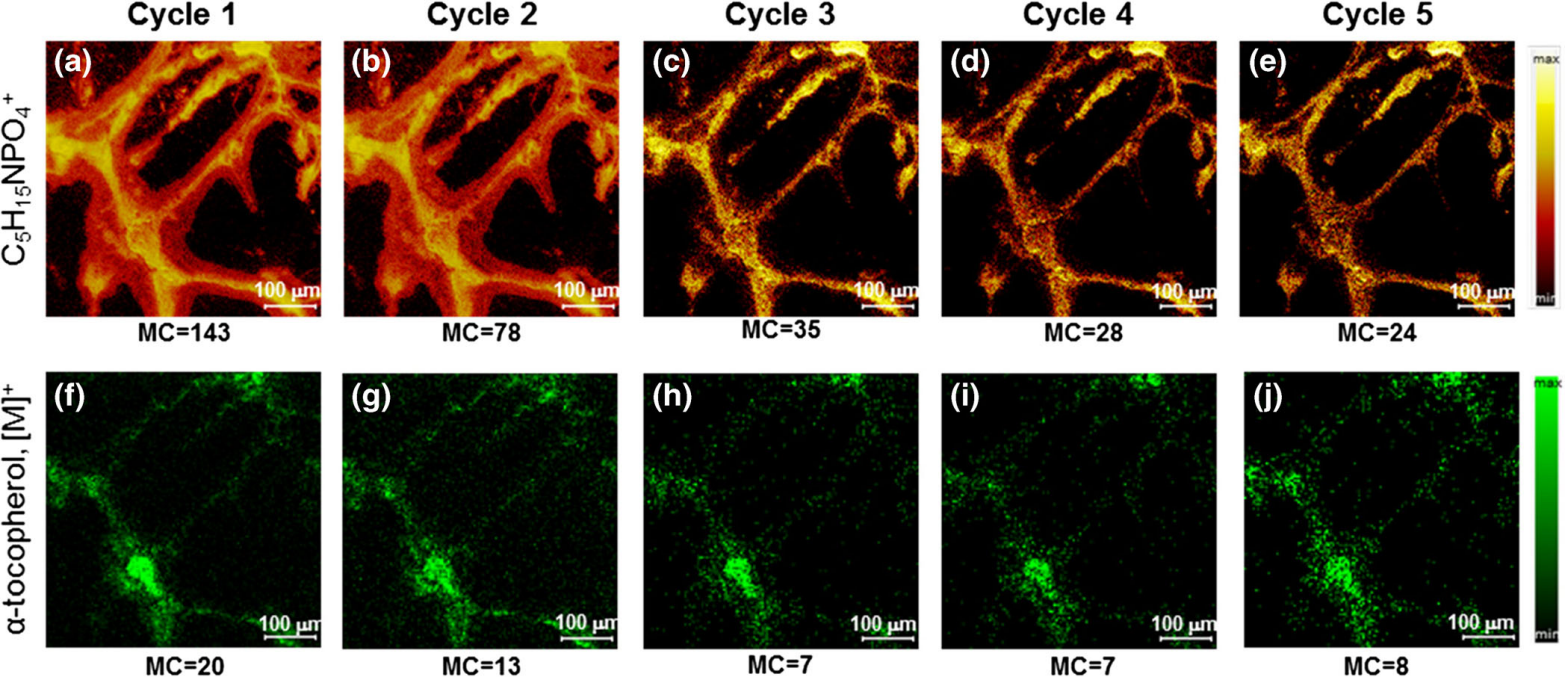
3D TOF-SIMS tandem MS imaging of the human iPSC-derived neurons in the positive ion mode. Each 2D image in a row was extracted from the 3D image volume. The top row displays MS^1^ images of the phosphocholine head group (C_5_H_15_NPO_4_^+^), and the bottom row displays MS^2^ TIC images of a molecule identified as the [M]^+^ of α-tocopherol, as a function of sputtered depth. The MS^1^ and MS^2^ data were collected simultaneously. The maximum counts per pixel are given in each panel. The soma and neurites of the cell are clearly observed in both the MS^1^ and the MS^2^ images

The in-depth analysis consisted of 5 analysis cycles where the surface was interrogated by ions from the Bi_3_^+^ LMIG, and 4 sputter cycles where surface material was removed using ions from an Ar-GCIB. The ion distribution of phosphocholine (MS^1^ *m/z* 184) and α-tocopherol (MS^2^ TIC) per cycle are plotted in Fig. 3. The spatial resolving power of the ion images was evaluated and calculated as the distance between 20 and 80% intensity of a feature within the image. This value can differ within the same image, due more or less to the sharpness at the edges between different features (i.e., the difference between cell body and neurites), but for both MS^1^ and MS^2^ images, the spatial resolution is measured to be less than a micron, and for more distinct features approximately 500 nm.

The Ar-GCIB sputtering is said to be non-destructive meaning that during removal of molecular layers from the surface, there is no degradation of the molecules via ion-induced fragmentation. We can say nothing of the possibility of differential sputtering but we assume the sputtering of organic molecules from the cells, whatever their composition, is nominally uniform. It appears from the first three panels in Fig. 3(a-c) that the lipid residue associated with the outer layer of the cell surface was removed during the first 2 sputter cycles. The residue ostensibly originated from delocalization of either the cell membrane during washing with an ammonium acetate buffer or during the freeze-drying, or from the cell culture medium. Although the Si wafer substrate containing the cells was blotted to remove excess buffer solution after being washed, it is inevitable that a small amount of liquid remains around the cells that could have caused such observed delocalization.

The maximum counts for the most intense pixels are noted on each image panel. These images demonstrate that the signal of both MS^1^ and MS^2^ decreases after the first sputter cycles (Fig. 3(a–c, f–h)) and then remains stable for successive analysis/sputter cycles (Fig. 3(c–e, h–j)). The clear presence of α-tocopherol after 4 sputter cycles indicates that surface debris from cell culture medium was removed in the first 2 measurement cycles and that there is plenty of signal, even within a single cycle, for reasonable contrast in the tandem MS images. This in-depth analysis was pre-set to conclude after 4 sputter cycles because it was thought that the cells would have been sputtered through based on the calibrated sputter rate determined using poly(methyl methacrylate) (PMMA) thin films. Clearly, this was not the case. It is likely that the inorganic buffer salts present at the surface significantly reduced the sputter rate. We observe that the sputtering has barely penetrated through the neurites, which are thought to be only 100–300 nm in diameter, by the last analysis cycle.

**Figure 4:**
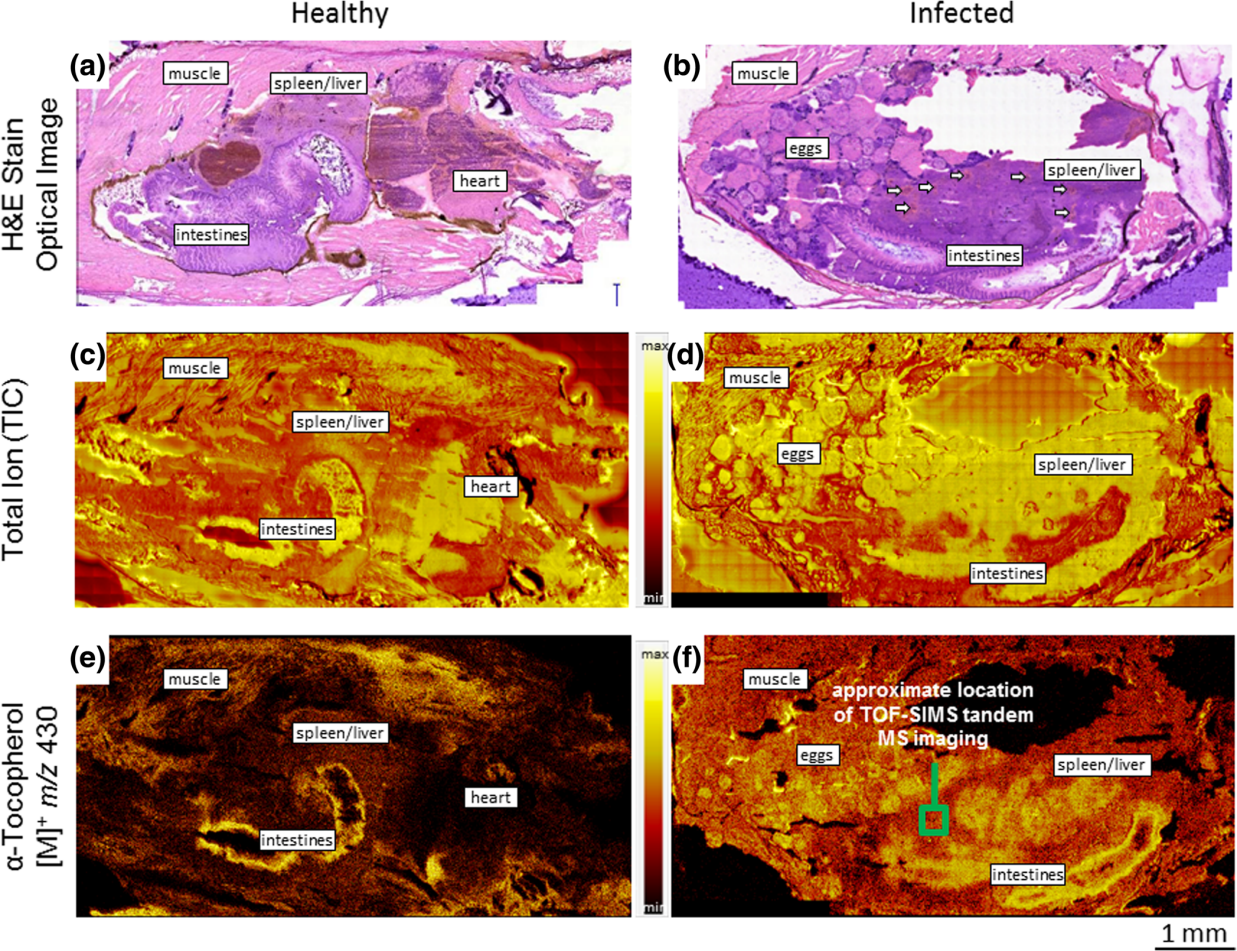
Histology of hematoxylin and eosin (H & E) stained sections from the abdomen of a healthy uninfected adult zebrafish (**a**) and a diseased adult zebrafish infected by *M. marinum* (**b**), showing severe granulomatous infection. The granulomas are visible in the H & E stain image as lighter structures with clearly visible cores which are indicated with small white arrows. TOF-SIMS (MS^1^) total ion current (TIC) images, acquired in the positive ion polarity, of healthy (**c**) and diseased (**d**) zebrafish tissue sections. The sections used for MS imaging analysis were consecutive sections adjacent to those used for histology. The major organ groups are identified. TOF-SIMS (MS^1^) images of healthy (**e**) and diseased (**f**) zebrafish tissue sections produced from positive polarity ions at *m/z* 430 which were identified by tandem MS (MS^2^) imaging as α-tocopherol. The green box in panel (**f**) indicates the approximate location of the tandem MS analysis for identification of the *m/z* 430 precursor ions

Two TOF-SIMS imaging data sets were generated of the abdominal area of zebrafish that were part of a study in which the fish were used as a model for tuberculosis. One of the tissue sections originates from a healthy control, while the other one was infected with *M. marinum,* in which severe granulomatous inflammation in the spleen and liver area was observed. This diagnosis is based on the optical images of hematoxylin and eosin (H & E) stained sections that were sampled adjacent to the sections subjected to TOF-SIMS analysis. These histological images are presented in the top row of Fig. 4, wherein the intestinal and spleen and liver areas of both the healthy control and the infected fish are labeled. There are no abnormalities observed in the spleen and liver of the healthy fish, while the corresponding area in the infected fish is full of granulomas which are visible as lighter structures. Further, some granulomas have necrotic cores, visible as darker or reddish spots, of which the biggest ones are indicated by small white arrows in Fig. 4(b).

In the search for differences between the healthy control versus the infected and diseased fish, *m/z* 430 was found to display a distinctive distribution. The spatial distributions of *m/z* 430 obtained by TOF-SIMS imaging of both samples are visualized in Fig. 4(e–f). The corresponding total ion (TIC) images are also provided in Fig. 4(c–d). The *m/z* 430 images demonstrate a greater expression of signal in the granulomatous areas within the spleen and liver of the infected fish section. It is postulated that this observation is an indication of a potential α-tocopherol-mediated antioxidant response by the host cells to the disease-induced inflammatory damage [7]. The *m/z* 430 peak was targeted for TOF-SIMS tandem MS imaging to confirm its identity. The precursor selection was centered at *m/z* 430.20, and the analysis was conducted in a 300 μm × 300 μm FOV. The approximate analysis area is identified by the inset in Fig. 4(f), adjacent to the granulomatous infection area but still within the region of the liver and spleen. Because the primary ion dose density is typically very low, e.g., on the order of 10^10^ ions/cm^2^, a tissue section can be sampled relentlessly which provides the possibility to identify dozens of molecular precursors by tandem MS analysis. In the case of the *m/z* 430 identification on the diseased zebrafish tissue section, the ion dose was much higher at 2.73 × 10^12^ ions/cm^2^; however, the molecular signals (i.e., molecular peaks at *m/z* > 300) plotted versus time show absolutely no degradation. The molecular signals were observed to increase between 1.5×–3× indicating that the surface of the tissue section had a layer of adventitious contamination that was expelled as the analytical ion dose increased. Numerous TOF-SIMS tandem MS imaging analyses were subsequently conducted on this same tissue section, including whole section imaging, and the high ion dose imparted to the 300 μm × 300 μm area utilized for *m/z* 430 identification was not specifically observable.

The MS^1^ precursor ion spectrum and the MS^2^ product ion spectrum of the 300 μm × 300 μm analysis area are provided in Fig. 1(d) and (e), respectively. The MS^1^ spectrum is perturbed in the vicinity of 3–4 Da on either side of the monoisotopic precursor selection window, centered at *m/z* 430.20, as illustrated by the Fig. 1(d) inset. The product ion peaks used for identification of the precursor ions by reference library matching include those at *m/z* 205, 191, 177, 165, 149, 136, 121, 71, 57, 43, and the *m/z* 430 precursor. Positive identification of the *m/z* 430 precursor as α-tocopherol was achieved using the both the Scripps Metlin MS/MS metabolite database and the NIST MS/MS reference library. Next, putative structures were assigned to the characteristic product ions as revealed in Fig. 1(e); the product ions at *m/z* 71, 57, and 43 have compositions of C_5_H_11_^+^, C_4_H_9_^+^, and C_3_H_7_^+^, respectively. Further confidence in the precursor identification was achieved by exact mass following calibration of the MS^2^ spectrum using the putative peak assignments. The resulting mass accuracies (Δ_*m/z*_) were measured to be −1.95 ppm across the product ion spectrum and −0.80 ppm at the precursor. Rather conclusively, and within a mass error of 0.00034 Da, the *m/z* 430.20 precursor ions observed in the diseased zebrafish have been identified as α-tocopherol [M]^+^ (C_29_H_50_O_2_^+^) ions.

Because the analysis time was of greater duration, the product ion spectrum of the α-tocopherol is of better fidelity in this analysis which allowed the observation of collisionally activated dissociation at every C-C bond. There are only rare cases when multiply charged secondary ions are observed in TOF-SIMS; therefore, the precursor ion is always assumed to have a single charge. Consequently, any product ion observed in the MS^2^ spectrum must arise by a neutral loss from the precursor ion. We also consider that the probability of multiple collisions or significant rearrangement products is negligible because the MS^2^ duty cycle is a mere 52 μsec and the transit time of precursor ions through the collision cell is less than one microsecond (< 1 μsec). Given such boundary conditions, the identification of product ion peaks is relatively straight forward.

From the precursor, we observe nominal mass losses of 18 and 28 Da corresponding to neutral loss of H_2_O or C≡O via distinct fragmentation channels. There is also a nominal mass loss of 56 Da from the precursor corresponding to neutral loss of H_2_C=C(CH_3_)_2_, isobutene. From this point, at *m/z* 374, there are a series of scissions at every C-C bond along the aliphatic chain of the α-tocopherol giving rise to peaks separated by 14 Da (CH_2_ loss) and 16 Da (CH_4_ loss) and ending with C_10_H_13_O_2_^+^ (*m/z* 165) which is the most prominent product ion in the spectrum. From the ring structure at *m/z* 165, we observe methylene, methane, acetylene, ethylene, oxygen, and water losses. A series of prominent hydrocarbon fragments at *m/z* 43, 57, and 71, having compositions of C_5_H_11_^+^, C_4_H_9_^+^, and C_3_H_7_^+^, respectively, are observed. The lowest observed product ion is C^+^ (*m/z* 12). Accordingly, we are able to unambiguously establish the identification of α-tocopherol via observation of the complete molecular structure. Remarkably, the robustness of the MS^2^ molecular identification is only weakly related to the MS^1^ performance. We have described elsewhere that the MS^1^ spectrum may have only unit mass resolution and accordingly poor mass accuracy, but the MS^2^ spectrum acquired in parallel will be produced with full mass resolution and mass accuracy [26]. Here, we detail in two different experiments the tandem MS identification of α-tocopherol, with precursor ions originating at *m/z* 430.40 and *m/z* 430.20 in the MS^1^ spectrum, and in each case providing the same result with high confidence.

Elevated levels of fatty acids were observed in the same areas on the same tissue section in addition to the increase of α-tocopherol in the infected areas, as described in other reports [30]. This is consistent with the findings described by Neyrolles et al. [31], where the increase of fatty acids is the result of hydrolysis of host cell lipids by the mycobacteria which take up hydrolysed lipids as a new fuel source [32]. We found evidence in this same study, measured at a lateral resolution (Δl) < 1 μm and reported elsewhere, which supports such a hypothesis [30].

## Conclusion

We have shown that the uncertainty of molecular identification in the analysis of complex biological samples can be significantly reduced or removed with the utilization of TOF-SIMS tandem MS imaging. Heretofore, the difficulties of biological analysis lie in the limitations of the employed MS imaging technique, namely, in the lack of an identification capability necessitating ex situ or parallel analyses, the time of MS imaging analysis which can be onerous, or in the prospect of sample consumption during analysis which forced the analyst to select which molecules of importance were targeted for identification. We have demonstrated here via 2D and 3D TOF-SIMS tandem MS imaging molecular identification can be accomplished in situ, without sample consumption or undesirable degradation, and in a reasonable time frame. Moreover, no TOF-SIMS information is discarded while conducting a simultaneous tandem MS analysis.

Importantly, the demonstrated capability for TOF-SIMS tandem MS imaging can be applied to assess the localization of a vast range of membrane-associated, as well as intracellular molecules and lipids [33], providing a means to confidently establish molecular identification and to visualize those same molecules which was not previously possible, also improving spatial resolution over previously available techniques. Specifically, mapping the spatial distribution of a molecule like α-tocopherol in 2- and 3-dimensions as in this study, using both thin tissue sections and cultured cells, demonstrates a unique and valuable tool to investigate the role of oxidative stress in disease states.

## Electronic Supplementary Material

ESM 1 (DOCX 535 kb)
