# Supplementary material for: Identification and High-Resolution Imaging of α-Tocopherol from Human Cells to Whole Animals by TOF-SIMS Tandem Mass Spectrometry
Source: J Am Soc Mass Spectrom. 2018 Jun 12;29(8):1571–81. doi: 10.1007/s13361-018-1979-x (PMC6060986; doi:10.1007/s13361-018-1979-x)
Supplement: Supplementary file 1 — (DOCX 535 kb) [file 13361_2018_1979_MOESM1_ESM.docx]

**Identification and High Resolution Imaging of α-Tocopherol from Human Cells to Whole Animals by TOF-SIMS Tandem Mass Spectrometry**

Anne L. Bruinen^1^, Gregory L. Fisher^2^, Rachelle Balez^3^, Astrid M. van der Sar^4^, Lezanne Ooi^3^, Ron M.A. Heeren^1^

^1^M4i, the Maastricht MultiModal Molecular Imaging Institute, Maastricht University, Universiteitssingel 50, 6229 ER Maastricht, the Netherlands

^2^Physical Electronics, Inc., Chanhassen, Minnesota 55317, United States

^3^Illawarra Health and Medical Research Institute, School of Biological Sciences, University of Wollongong, Wollongong, NSW, 2522, Australia

^4^VU University medical center, 1081 HV, Amsterdam, the Netherlands


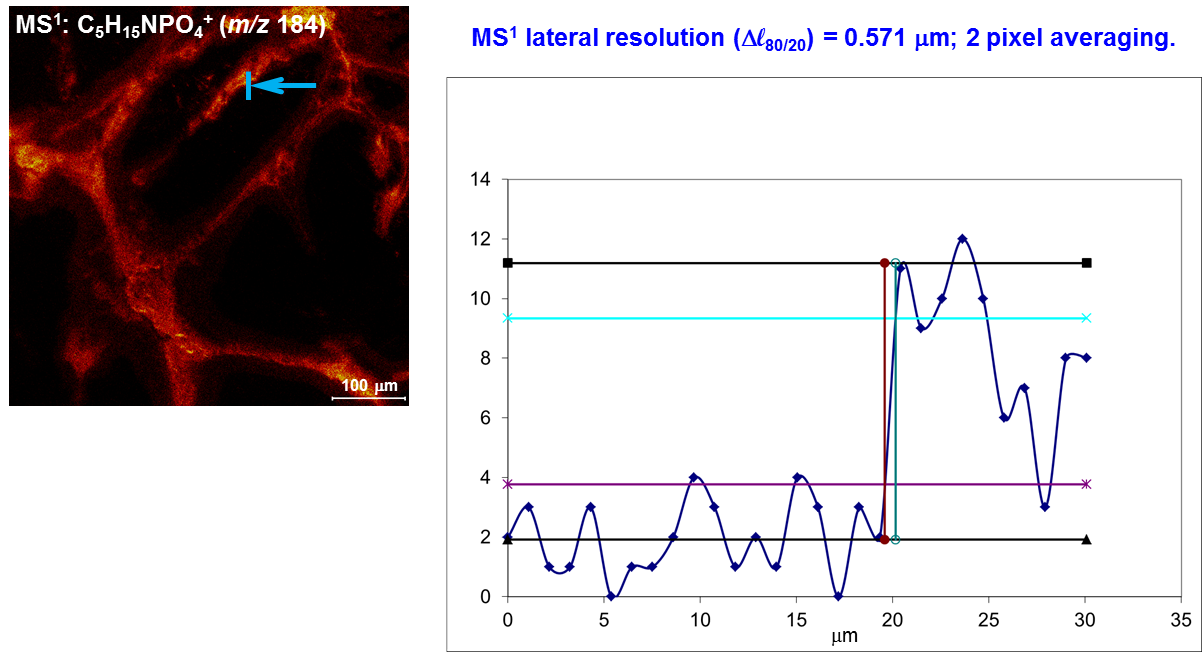


**Supplemental Figure 1:** MS^1^ map of the phosphocholine headgroup (C_5_H_15_NPO4^+^) at *m/z* 184 with a 550 μm x 550 μm FOV wherein a line scan measurement was made as indicated by the blue line. The line scan on the right shows a lateral resolution (Δl_80/20_) of 0.571 μm with 2 pixel averaging.


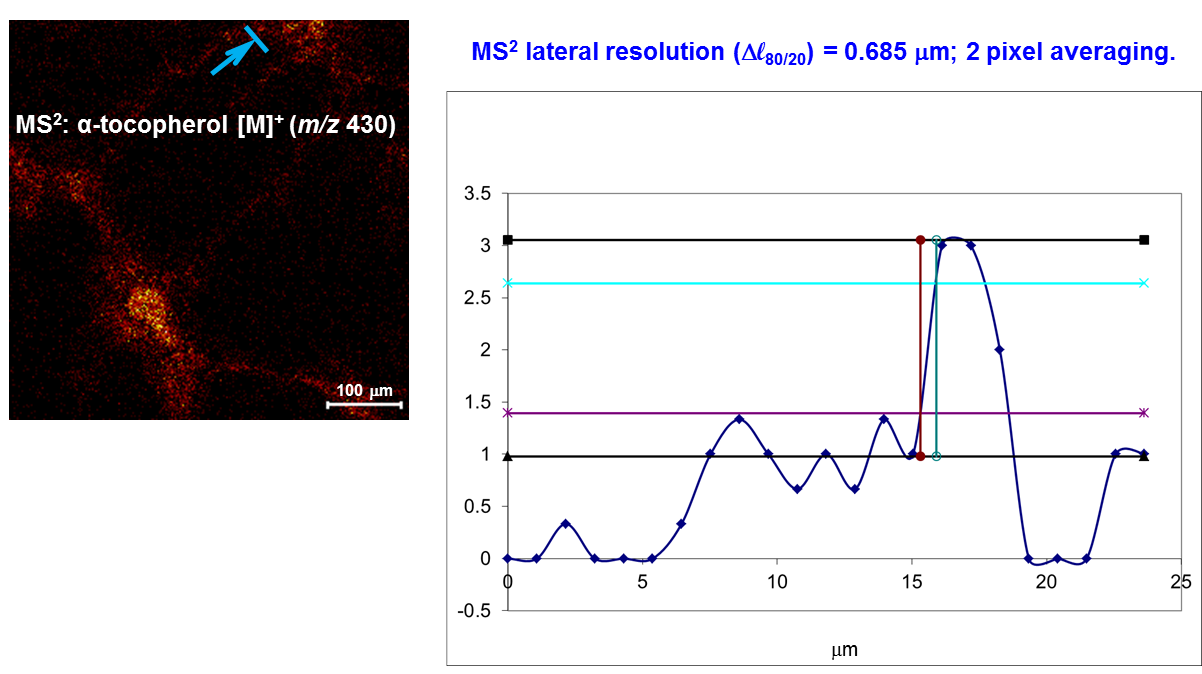


**Supplemental Figure 2:** MS^2^ map representing the total *m/z* 430 (α-tocopherol) signal acquired simultaneously with the image shown in **Supplemental Figure 1** with a 550 μm x 550 μm FOV wherein a line scan measurement was made as indicated by the blue line. The line scan on the right shows a lateral resolution (Δl_80/20_) of 0.685 μm with 2 pixel averaging.
